# Set back on the way to meet SDG targets: progress in tackling tuberculosis reversed by the pandemic 

**DOI:** 10.2807/1560-7917.ES.2022.27.12.2203241

**Published:** 2022-03-24

**Authors:** Eurosurveillance editorial team

**Affiliations:** 1 European Centre for Disease Prevention and Control (ECDC), Stockholm, Sweden

**Keywords:** tuberculosis, TB, epidemic

Each year on 24 March, World Tuberculosis Day provides an opportunity to capture progress as well as challenges on the way to ending the global tuberculosis (TB) epidemic as set out by the United Nations Sustainable Development Goals (SDG) [1]. 

Two years after the World Health Organization (WHO) declared the coronavirus disease (COVID-19) a pandemic [2], its impact on the response to other (infectious) diseases becomes more and more apparent. One example: for the first time since 2005, TB-related deaths increased worldwide in 2020 [3]. Data in the WHO Regional Office for Europe and European Centre for Diseases Prevention and Control TB surveillance report for the 53 countries of the WHO European Region published today also demonstrate that TB mortality plateaued in Europe overall between 2019 and 2020 for the first time in the last two decades, most likely as a result of undiagnosed and untreated TB due to disruptions in essential TB services caused by the COVID-19 pandemic [4]. 

This year’s World TB Day theme *‘Invest to End TB. Save lives.’* aims to remind public health decision-makers worldwide that achieving the SDG targets by 2030 requires constant commitment and investments to not jeopardise the progress of the last decades.

Marking World TB Day 2022, this issue of *Eurosurveillance* includes three articles illustrating persistent challenges in the TB response among certain population groups and screening approaches across Europe.

Erkens et al. [5] analysed risk factors of recurrent TB and distinguish reactivation and reinfection in patients in the Netherlands after they had completed or interrupted their treatment. Based on their findings from a study period covering 24 years, the authors suggest that TB patients should be monitored for 2─5 years following their treatment, irrespective if it was interrupted or not and they also recommend that there should be guidelines to support early detection of recurrent TB.

Conducting a nationwide retrospective register-based case–control study (1990 to 2018) in Denmark, Nordholm et al. [6] investigated risk factors for TB in pregnant women and those who had given birth. According to their data analysis, migrant women who had stayed in Denmark a median time of around 3 years had an increased risk of developing TB during pregnancy and after giving birth. The authors thus advocate for early case finding through targeted TB screening of selected pregnant women at risk to prevent TB among mothers and their newborn children. 

This corresponds to findings from Margineanu et al. [7] who suggest a renewed focus on ways to include migrants in national or local TB programmes if the TB elimination targets are to be met. Based on their survey results, just 15 of the 30 European Union and European Economic Area countries and Switzerland have screening programmes for latent TB infection in place that target migrants, while five additional countries plan to implement one in the near future.

On the occasion of World TB Day, we have updated our collection of TB articles.
